# Effect of Exercise on Sarcopenia among Cancer Survivors: A Systematic Review

**DOI:** 10.3390/cancers14030786

**Published:** 2022-02-03

**Authors:** Anlan Cao, Leah M. Ferrucci, Bette J. Caan, Melinda L. Irwin

**Affiliations:** 1 Department of Chronic Disease Epidemiology, Yale School of Public Health, 60 College Street, New Haven, CT 06520-8034, USA; leah.ferrucci@yale.edu (L.M.F.); melinda.irwin@yale.edu (M.L.I.); 2 Yale Cancer Center, New Haven, CT 06520-8034, USA; 3 Division of Research, Kaiser Permanente Northern California, 2000 Broadway, Oakland, CA 94612, USA; bette.caan@kp.org

**Keywords:** exercise, physical activity, sarcopenia, muscle mass index, cancer

## Abstract

**Simple Summary:**

Sarcopenia is a condition characterized by loss of skeletal muscle mass and low muscle strength or physical performance. Cancer survivors are likely to be impacted by sarcopenia and suffer from a worse prognosis. Exercise has been suggested to be a promising tool to attenuate sarcopenia, but its effect among cancer survivors has not been systematically tested yet. We conducted a systematic review of seven interventional studies examining the effects of exercise on sarcopenia among cancer survivors. Results suggested that exercise improved muscle quantity and potentially reversed sarcopenia among breast, gastric, prostate and liver cancer survivors. If the relationship is further supported by larger trials, we could potentially identify cancer survivors at higher risk of adverse health outcomes by screening for sarcopenia and improve their prognosis and quality of life through exercise interventions.

**Abstract:**

Sarcopenia is related to adverse health outcomes in cancer survivors. Previous reviews reported exercise improved muscle mass or function in cancer survivors, but thus far a systematic review examining the effect of exercise on sarcopenia in this population has not been conducted. Therefore, we systematically searched PubMed, CENTRAL (Cochrane Central Register of Controlled Trials) and ClinicalTrials.gov for publications and ongoing trials (through November 2021) that reported exercise interventions and diagnosed sarcopenia among cancer survivors. Seven exercise trials were eligible for this review. Six of seven studies showed exercise increased skeletal muscle post intervention (1.6% to 5.4% increase within intervention groups compared to baseline, *p* ≤ 0.07; 2.1% to 12.8% greater increase for intervention than control groups, *p* ≤ 0.02) and in the three studies that reported sarcopenia reversal, an improvement (18.2% to 42.9% decrease in sarcopenia in exercise groups, 5.2% increase to 16.7% decrease in sarcopenia in control groups, *p* = 0.04) was observed. Existing research indicates the potential for exercise to improve health outcomes for cancer survivors through building muscle and attenuating sarcopenia. More high-quality, long-term, large randomized controlled trials examining effects of different exercise types and doses to improve sarcopenia should be conducted to further explore this important topic.

## 1. Introduction

Cancer is the second leading cause of death worldwide [1]. In the United States in 2021, there was an estimated 1.9 million new cancer cases and 0.6 million cancer deaths [2]. Sarcopenia, a syndrome characterized by progressive and generalized loss of skeletal muscle mass and low muscle strength or physical performance [3], has been hypothesized to be a powerful predictor of cancer morbidity and mortality, including postoperative complications, treatment-related toxicities, fractures, falls, and prolonged hospitalization [4,5,6,7]. A meta-analysis of 38 studies found that sarcopenia was associated with a 44% higher risk of all-cause mortality in cancer patients with solid tumors (HR = 1.44, 95% CI: 1.32–1.56) [5]. 

Primary sarcopenia is associated with aging, and commonly seen in older adults [8]. Secondary sarcopenia results from advanced disease, insulin resistance, inadequate nutrition, or inactivity, such as bed rest [9]. According to analyses of the National Health and Nutrition Examination Survey (NHANES) 1999–2004, 9% of people in the general population older than 20 years in the United States are sarcopenic [10], and the sex-specific prevalence of sarcopenia in the adults older than 65 years is 28.5% and 18.9% among men and women, respectively (based on body mass index adjusted appendicular skeletal muscle) [11]. A previous review found sarcopenia prevalence ranged from 14% to 79% among cancer survivors [12]. The highest prevalence was found in patients with esophagogastric cancer (43% to 79%), pancreatic cancer (56% to 63%), liver cancer (28% to 68%), or renal cell carcinoma (53% to 54%) [12]. Since cancer risk increases with age [2], older cancer survivors are at higher risk for both primary sarcopenia and secondary sarcopenia due to cancer or its treatments. Therefore, it is vital to prevent and treat sarcopenia in cancer survivors, especially the older adults, to reduce adverse health outcomes and improve their prognosis.

A meta-analysis of observational studies found greater daily physical activity was associated with reduced odds of developing sarcopenia in older populations without cancer (OR = 0.45, 95% CI: 0.37–0.55) [13]. In community-dwelling older adults with sarcopenia, a meta-analysis suggested exercise increased appendicular skeletal muscle mass, knee extension strength and walking speed following 3 months of intervention [14]. Another systematic review and meta-analysis of 22 trials found improved muscle strength and physical performance with exercise in a general population, though differences in muscle mass were limited [15]. Additionally, previous systematic reviews indicated resistance exercise increased muscle mass and function in cancer survivors [16,17], and aerobic training had a significant impact on muscle strength and physical function in a study of breast cancer survivors [18], but whether the increase was clinically significant remains unknown. 

The American Cancer Society recommends 2.5 h of moderate-intensity exercise per week and twice-weekly resistance training for cancer survivors [19], in part because of exercise’s role in improving quality of life, physical function, arthralgia, mental health, and cancer-related fatigue [16,20,21]. Despite exercise being a viable mechanism to affect sarcopenia and exercise’s important role in cancer outcomes, to our knowledge there has not been a systematic review examining the impact of exercise interventions specifically on sarcopenia among cancer survivors. Therefore, we aimed to systematically examine if there are benefits from exercise among cancer survivors in the domain of sarcopenia, which could improve morbidity and mortality outcomes in these individuals.

## 2. Methods

This systematic review adhered to the Preferred Reporting Items for Systematic Reviews and Meta-Analyses (PRISMA) guidelines and was registered with PROSPERO (registration number: CRD42021237010).

### 2.1. Search Strategy

**Figure 1:**
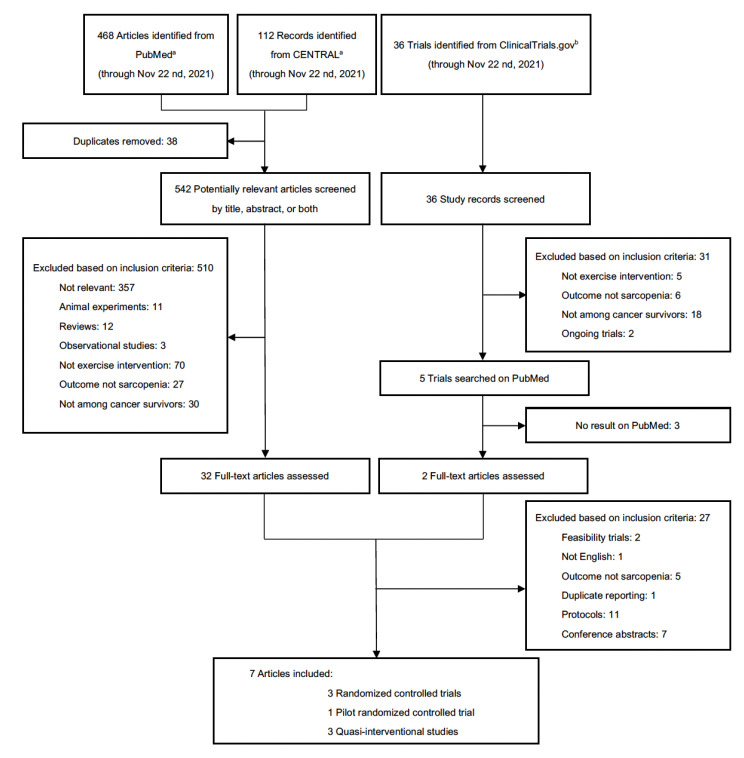
Selection process of included studies. ^a^ PubMed and CENTRAL (Cochrane Central Register of Controlled Trials) search terms: ((exercise) OR (exercises) OR (physical activity) OR (physical activities)) AND ((sarcopenia) OR (sarcopenic obesity)) AND ((cancer) OR (neoplas*) OR (tumor)). ^b^ ClinicalTrials.gov search terms: Condition or Disease: (sarcopenia) OR (sarcopenic obesity); Intervention/treatment: (exercise) OR (exercises) OR (physical activity) OR (physical activities); Other terms: (cancer) OR (neoplas*) OR (tumor); Study type: Interventional studies (Clinical Trials).

We conducted a systematic search on PubMed, CENTRAL (Cochrane Central Register of Controlled Trials) and ClinicalTrials.gov from inception until November 2021 using keywords related to “exercise”, “physical activity”, “sarcopenia” and “cancer”, resulting in 468 studies from PubMed, 112 records from CENTRAL and 36 trials from ClinicalTrial.gov. Details of the search strategy, keywords, and selection process are shown in Figure 1. 

### 2.2. Inclusion and Exclusion Criteria

Inclusion criteria were as follows: (a) exercise intervention; (b) clinically defined sarcopenia described in study (e.g., enrollment criteria, description of study population); (c) sarcopenia or muscle mass as the primary or secondary outcome; (d) participants were cancer survivors; (e) reported study results; (f) written in English. Randomized controlled trials (RCT), pilot studies and quasi-experimental studies were all eligible. The interventions could contain any form of physical activity (e.g., aerobic training, resistance training, combination). Cancer survivor was defined as anyone diagnosed with cancer. Studies that only included lean body mass, muscle mass or physical function measures, but did not specifically describe clinically defined sarcopenia, were excluded. 

Since there are no standard diagnostic criteria for sarcopenia, any diagnostic criteria were eligible. Skeletal muscle index (SMI) and appendicular skeletal muscle index (ASMI), calculated as lean body mass, appendicular lean body mass measured by dual-energy X-ray absorptiometry (DEXA) or bioimpedance analysis (BIA), or muscle mass measured by computer tomography (CT), and divided by square of height, are well-recognized as measures of muscle quantity [3]. Total lean body mass (LBM) measured via DEXA was also included. Grip strength or chair stand test are sensitive indicators for muscle strength [3]. Low gait speed (GS) is commonly used to reflect limited physical function [3]. 

### 2.3. Selection Procedure

Two authors (A.C. and M.L.I.) independently assessed study eligibility by screening PubMed and CENTRAL abstracts or Clinicaltrials.gov records. Final eligibility of the selected abstracts was determined via full-text review by one author (A.C.) and verified by all co-authors. For ClinicalTrials.gov records, detailed study records were screened. Potentially relevant studies were searched on PubMed according to keywords, investigators and trial registration numbers and identified studies were assessed for eligibility. 

### 2.4. Data Extraction

A standard spreadsheet was used to extract data on study design, participants and eligibility, intervention design, outcome definition and measurements, study duration, adherence to intervention, changes in sarcopenia status and other main findings. 

### 2.5. Risk of Bias Assessment

Included studies were evaluated for risk of bias using Version 2 of the Cochrane risk-of-bias tool for randomized trials (RoB 2) [22]. The following domains of each included study were assessed: randomization process, deviations from the intended interventions, missing outcome data, measurement of the outcome, and selection of the reported result. Specifically, for domain “deviations from the intended interventions”, two-arm trials were assessed for the effect of assignment to the interventions, and single-arm experimental studies were assessed for the effect of adhering to intervention. A risk of bias summary plot was generated using RevMan 5.4.1.

### 2.6. Data Synthesis

Difference between change in muscle mass (index) in the exercise arm and the comparison arm of each study was converted into percentage to allow for comparison between studies; no quantitative synthesis was conducted. The comparison groups of the included studies were usual care alone or a combination of usual care and another kind of exercise. A narrative synthesis of all study results discussing the overall effect of exercise on muscle mass and sarcopenia among cancer survivors was conducted. 

## 3. Results

After removing duplicates, 542 abstracts from PubMed and CENTRAL, and 36 trial records from ClinicalTrials.gov were screened, and 510 studies and 31 trial records were excluded based on inclusion criteria (Figure 1). Among the 34 full-text studies reviewed, seven exercise trials were considered eligible [23,24,25,26,27,28,29]. 

Among the seven included studies, four were RCTs [23,25,26,28], though one of these was a pilot study [25]. The other three were quasi-experimental studies; two were single-cohort experimental interventions [24,29] and one was a non-randomized trial [27].

### 3.1. Measurement and Diagnostic Criteria for Sarcopenia

Three studies measured total lean body mass or appendicular lean mass via DEXA, and further estimated SMI or ASMI [23,25,26] (Table 1). Three studies employed CT to estimate muscle mass, two of them calculated SMI [27,29], and one used total psoas index (TPI) [28]. Finally, Yamamoto et al. used BIA measured muscle mass, handgrip strength and GS for sarcopenic status of participants [24]. The cut-points for sarcopenia diagnosis for each study also varied.

### 3.2. Baseline Characteristics of Study Participants

The studies had relatively small sample sizes (ranging from 22 to 209) and participants were middle-aged or older cancer survivors (mean age: 48.8 to 75.0) (Table 2). The baseline prevalence of sarcopenia ranged from 14.0% to 100%. Breast [23,26,29], gastric [24], prostate [25], hepatocellular [27] and rectal [28] cancer survivors were enrolled in these studies. Two of the breast cancer studies [23,26] only included early stage patients, while Delrieu et al. enrolled women with metastatic breast cancer [29]. The studies varied in terms of treatment status. Participants were scheduled to have surgery in two studies [24,28] while participants were receiving neoadjuvant chemotherapy in the Moug et al. trial [28]. Two studies had patients receiving adjuvant chemotherapy or radiotherapy [23,29] and another involved patients receiving androgen deprivation therapy [25]. The last two studies enrolled participants that had completed chemotherapy and/or radiotherapy, but were within 6 months of treatment completion [26] or had already gone through transcatheter arterial chemoembolization [27].

### 3.3. Intervention Design

Three studies had combined resistance and aerobic exercise [24,26,27]; one was supervised by a certified American College of Sports Medicine/American Cancer Society (ACSM/ACS) cancer exercise trainer [26], one was conducted in-hospital [27], and one was unsupervised home-based exercise [24] (Table 3). One study employed resistance exercise supervised by an accredited exercise trainer [25], and the other study had two exercise groups and compared resistance training to aerobic training (also supervised, but did not specify the type of trainer) [23]. Two more recently published studies employed unsupervised aerobic exercise training, mostly brisk walking [28,29]. The longest intervention period was 6 months [29], while the shortest study was 16 days [24]. Most trials were between two and three months long. The adherence was unsatisfactory in Yamamoto’s study (50%) [24], but the other studies had fair to good adherence, ranging from 68% to 95%. 

### 3.4. Change in Muscle Index and Sarcopenia Reversal

Six of the seven exercise trials increased SMI, ASMI or TPI in the intervention groups at the end of the studies, one study did not find improvement in muscle mass, and three studies reported the effect of exercise on sarcopenia reversal (Table 3).

Among randomized exercise trials, Adams et al. [23] found significantly higher post-intervention SMI in the resistance training group compared to the usual care group (2.1%, 0.32 kg/m^2^, 95% CI: 0.04–0.60 kg/m^2^). Dieli-Conwright et al. [26] reported a 12.8% increase in LBM in the exercise group compared to the usual care group (between-group difference in post-intervention LBM = 7.7 kg/m^2^, 95% CI: 5.5–10.3 kg, *p* = 0.001). ASMI was also reported in the study, with a 50% increase in ASMI in the exercise group compared to the usual care group (between-group difference in change of ASMI = 2.4 kg/m^2^, 95% CI: 1.3–4.1 kg/m^2^). Dawson et al. [25] also observed a significant increase in ASMI in the exercise groups compared to the non-exercise groups (3.7%, adjusted between group mean change = 0.3 kg/m^2^, 95% CI: 0.1–0.5 kg/m^2^). Moug et al. [28] found increased TPI in the exercise group and a decrease in TPI in the control group, although the difference was not statistically significant (+ 16.0 vs. −8.4 mm/m^2^, *p* = 0.07). After adjusting for age, comorbidities and baseline TPI, the group difference was 40.2 mm/m^2^ (*p* = 0.07). Three of these randomized exercise trials found significantly increased muscle mass or muscle index in the exercise arm post-intervention compared to baseline [23,25,26] (Table 3). 

As for sarcopenia reversal in RCTs, among 21 participants who were sarcopenic at baseline in the resistance training group in the Adams et al. study [23], nine (42.9%) were non-sarcopenic after the intervention compared to 16.7% sarcopenia reversal in the usual care and aerobic training groups (*p* = 0.039). Dawson et al. [25] observed a significant reduction in sarcopenia prevalence after the exercise intervention in the exercise groups compared to non-exercise groups (−23.1% vs. +5.2%, *p* = 0.04). 

The three quasi-experimental studies had similar results. Yamamoto et al. [24] found significantly improved handgrip strength compared to baseline (1.6%, 1.2 kg, *p* = 0.02). SMI and GS also increased, but were only borderline significant (*p* = 0.06). A total of 4 of the 22 participants (18.2%) with sarcopenia were no longer sarcopenic after the program. Koya et al. [27] found that SMI change was significantly higher in the exercise group than the control group (*p* < 0.01), although they did not report the effect size. Delrieu et al. [29] found significantly improved quadriceps strength and 6 min walking distance compared to pre-intervention (*p* < 0.001), but there were no significant changes in muscle characteristics including skeletal muscle radiodensity (*p* = 0.07) and skeletal muscle gauge (*p* = 0.06). 

### 3.5. Risk of Bias Assessment

**Figure 2:**
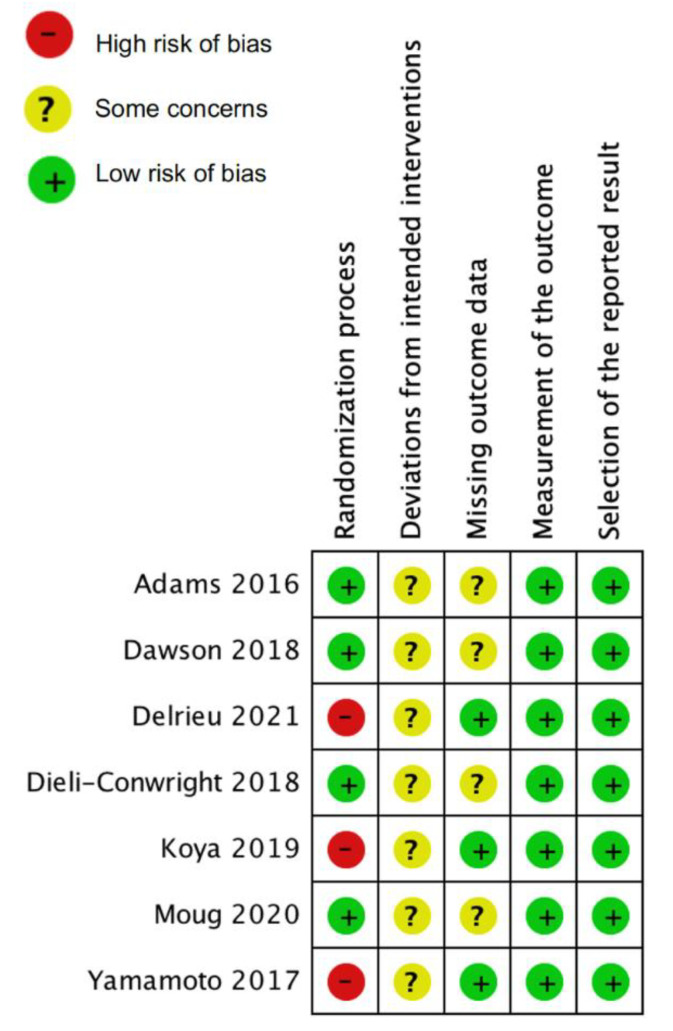
Risk of bias of included studies. Outcome assessed was: SMI for Adams et al. [23], Yamamoto et al. [24] and Koya et al. [27]; ASMI for Dawson et al. [25] and Dieli-Conwright et al. [26]; TPI for Moug et al. [28]; LBM for Delrieu et al. [29].

The overall risk of bias of included studies was of some concern, mainly due to potential bias arising from “deviations from intended intervention” and “missing outcome data.” (Figure 2) The three quasi-experimental studies were at high risk of bias due to the lack of randomization [24,27,29]. In addition, because participants in exercise trials cannot be blinded to intervention assignment, all studies included were judged as “some concerns” for “deviations from intended intervention.” The outcome availability in all the RCTs was less than 95% of the randomized participants. This was likely due to these outcomes being secondary analyses [23,25,28], except for Dieli-Conwright et al. [26]. Therefore the “missing outcome data” was classified as “some concerns” for the four RCTs. All included studies were at low risk of potential bias in measurement of the outcome and result reporting.

## 4. Discussion

This systematic review of the effect of exercise interventions on sarcopenia among cancer survivors found that exercise was effective in increasing SMI [23,24,27], ASMI [25], or LBM [26], improving muscle strength [24] and reversing sarcopenia [23,24,25]. However, increases in muscle mass were not statistically significant among late-stage breast cancer patients [29] or among patients in pre-habilitation [24,28]. It is important to note that Yamamoto et al. [24] and Delrieu et al. [29] were single-arm exercise trials without comparison groups prone to higher risks of bias, so these null results should be interpreted with caution. While overall there were a limited number of studies with data specific to sarcopenia, these findings are relevant for the prognosis and survival of cancer survivors, especially the older adults [30], as the prevention and/or reversal of sarcopenia via exercise interventions might be an approach to improve cancer outcomes in this population. 

The majority of exercise trials reported increased muscle mass (SMI, ASMI, LBM, TPI) in the intervention groups. Compared to baseline, a 1.6% to 3.6% increase in muscle index was observed after intervention [23,24,25,28]. Similarly, participants in the intervention groups of three studies had 2.1% to 6.9% higher muscle index or muscle mass than control groups upon study completion [23,25,28]. Despite the concerns for bias arising from not being able to mask participants and missing outcome data due these outcomes being secondary analyses, these studies provided reliable evidence supporting the effect of exercise in improving muscle mass. Improvement in the muscle index in the exercise group compared to the control group in general population samples (0.21 kg/m^2^, 95% CI: −0.05–0.48 kg/m^2^) [15] was similar to the effects in cancer survivors observed in Adams et al. (0.32 kg/m^2^) and Dawson et al. (0.3 kg/m^2^). Of note, the increases for all of the above reported measures in the Dieli-Conwright study (32% increase post intervention in the exercise group and a 2.5 kg/m^2^ (50%) increase in ASMI in exercise group compared to controls) are extreme outliers, have not been replicated in other studies of either cancer survivors or non-cancer survivors, and should be interpreted with caution [26]. The magnitude of SMI change compared to baseline was the lowest in Yamamoto et al. (1.6%) [24] and could stem from unsupervised exercise, low adherence (50.0%), or short intervention period (median = 16 days) due to the limited time window before surgery. Participants in the Yamamoto et al. study were severely sarcopenic and may also have had greater physical barriers to following the exercise guidance. Although nutritional support was also administered in this trial, a previous study indicated nutritional supplementation has limited effect in addition to resistance training on sarcopenia [31]. Koya et al.’s study was not randomized, so although the comparison groups were similar at baseline except for age, other potential confounding may exist [27]. Furthermore, since no effect size was reported, we cannot make quantitative comparisons to other studies [27]. An increase in TPI found by Moug et al. [28] was borderline significant (*p* = 0.07), although the effect size was relatively large (40.2 mm^2^/m^2^), the study could be underpowered due to a small sample size (N = 44). Delrieu et al. [29] did not observe any significant change in muscle characteristics after 6 months; however, given that the participants were at higher risk of muscle wasting due to metastatic cancer, the preservation of muscle mass should be acknowledged. 

Among the three studies that assessed reversal of sarcopenia, effect sizes ranged from 18.2% to 28.3% after the intervention [23,24,25]. Dawson et al. [25] observed the largest effect and could be explained by high adherence ( > 85%). Adams et al. pooled the aerobic exercise and usual care groups together, potentially underestimating the influence of the overall exercise intervention [23]. There was an 18.2% reversal of baseline sarcopenia in Yamamoto et al.’s study, but without a control group we cannot ascertain an unbiased effect estimate of short-term, pre-operative exercise intervention [24]. 

Cancer treatment can lead to significant muscle wasting through the suppression of appetite, the provoking of activation of NF-κB and the ubiquitin proteasome pathway [32], resulting in increased difficulties in preventing and treating sarcopenia. Adams et al. and Dieli-Conwright et al. both enrolled breast cancer survivors and the intervention frequencies (three times/week) and durations were similar (17 weeks vs. 16 weeks) [23,26]. The effect size in Dieli-Conwright et al. was larger than Adams et al. (change in muscle index: 50.0% vs. 2.1%) [23,26]. Besides differences in exercise structure, participants in Dieli-Conwright et al. had completed chemotherapy [26], while the Adams et al. subjects were receiving treatment [23]. Although previous studies suggested exercise can reduce cancer treatment side effects [33], treatment-related toxicities and side effects could contribute to the smaller effect of exercise in Adams et al. However, of note the magnitude of the effect size in the Adams study is much closer to the effect size from other studies included in this review. Aging is also a strong predictor of muscle loss. Four of the included studies recruited older participants (mean age: 63.7 to 75.0) [24,25,27,28], while three enrolled young to middle-age cancer survivors (mean age: 48.8 to 55) [23,26,29]. Higher baseline sarcopenia prevalence was seen in the older study populations [24,25] and higher cancer stage patients [29]. Due to the heterogeneity of cancer type of the included studies, we are not able to assess the possible interaction between cancer treatment and aging. Therefore, additional research on a range of cancer types and treatments is vital to evaluate such relationship in the domain of sarcopenia to better help older patients preserve muscle mass during and after cancer treatment. 

In addition to treatment related factors, inactivity and malnutrition, sarcopenia among cancer survivors could also be attributed to loss of α-motoneuron, increased interleukin-6 (IL-6) and blunted secretion of growth hormone [34,35]. The effect of exercise on sarcopenia could be due to its role in altering α-motoneuron properties [36] and impacting muscle fibers through IL-6 and growth hormone [37,38]. Future studies may be able to assess additional relevant biomarkers to tease out these potential mechanisms of action. 

Despite the heterogeneity of the included studies, significant improvements in muscle mass indices were observed in four trials [23,25,26,27], while two trials with borderline significant improvements could be underpowered due to sample size [24,28], and one study among late-stage patients indicated the potential of exercise in preserving muscle mass. In addition, reversal of sarcopenia was indicated in three of the studies [23,24,25]. The positive findings indicate preventing muscle loss during cancer treatment and recapturing muscle loss afterwards are feasible, which could significantly improve the prognosis of numerous cancer survivors.

### 4.1. Diagnosis and Measurements of Sarcopenia

It is important to note that the diagnostic criteria of sarcopenia varied among the included studies and in turn limited our ability to directly compare findings across these interventions. According to the latest recommendation of the European Working Group on Sarcopenia in Older People 2 (EWGSOP2), sarcopenia should be diagnosed when low muscle strength and low muscle quantity or quality are both presented, and an additional low physical performance would suggest that sarcopenia is severe [3]. The shift from using just muscle mass as the criterion for sarcopenia [9] to adding muscle strength as an indicator [3] is because muscle strength has been shown to be associated with adverse health outcomes, such as falling and fractures [39]. Moreover, the relationship between muscle mass and muscle strength is not linear, so the change of either cannot fully reflect the associated functional limitations or mortality risks [40]. The combination of them would be more informative and may have better predictive value for cancer prognosis; yet many organizations consider muscle mass to be the core component in defining sarcopenia [41,42,43]. 

Most of the included publications only used muscle index to define sarcopenia [23,25,26,27,28,29], and the cut-off points were not concordant with recommended values (ASMI measured via DEXA <7.0 kg/m^2^ for men and <5.5 kg/m^2^ for women) [3]. Specifically, Adams et al. defined class I sarcopenia as SMI lower than one standard deviation (SD) compared to the mean value [23], instead of 2 SD (for overall sarcopenia) as advised by EWGSOP2 [3]. Therefore, cancer survivors in this trial might have been less sarcopenic, and thus the sarcopenia could have been easier to reverse. Yamamoto and colleagues also included handgrip strength and physical function as a part of diagnostic criteria as recommended by EWGSOP2 [24]. Moreover, instead of using cut-off points to convert SMI/ASMI to a dichotomous variable (sarcopenic/not sarcopenic), Koya et al. treated SMI as a continuous variable [27], and other studies also examined the change of continuous SMI/ASMI before and after the exercise intervention [23,24,25,26,29]. Categorization allows for a clear definition of sarcopenia, but it could obscure the true dose–response relationship between SMI and prognosis. The current definition of sarcopenia implies that every woman with ASMI less than 5.5 kg/m^2^ has the same risk of death, yet people whose ASMI are 5.4 kg/m^2^ have significantly different risk than the ones with ASMI of 5.6 kg/m^2^. Therefore, future studies might consider SMI/ASMI as continuous variables to better reflect the risk associated with the loss of muscle mass in addition to diagnosis of sarcopenia. 

Investigators have used BIA, DEXA and CT to measure body composition, all of which have strengths and limitations. BIA is inexpensive and easy to administer, but it only measures the body’s resistance to a single or multiple electrical currents across a limb or through the body, which is strongly associated with total body water [44]. Therefore, the accuracy of BIA is limited by the high variance of the water volume of a person in different disease states. DEXA machines were initially designed to measure bone mineral density, and fat content can also be measured directly through differential absorption of two phyton energies, thus lean body mass can be determined by subtraction. Appendicular lean mass is used as a proxy for muscle mass but some studies have seen weak correlations with physical function [45]. CT provides a cross-sectional measurement of muscle area, though multiple CT scans can be used to estimate the volume of muscle or fat tissues directly. The muscle area from a single abdominal slice of a specific lumbar vertebral landmark (L_3_) is shown to be highly correlated with the total amount of muscle [46], thus CT is considered the gold standard for the non-invasive assessment of muscle quantity. However, strength training could have a major impact on muscle mass at limbs, which may not be reflected completely by a single slice at L_3_ level. In addition, the radiation exposure can be concerning to some participants, and the high cost impedes use of CT for large epidemiological studies. Recently, a novel method, D3-creatine dilution (D_3_-Cr), was established based on the assumption that 98% of the total body creatine pool is in skeletal muscle, where the creatine is turned into creatinine [47]. By administering a tracer dose of deuterated creatine (D_3_-Cr) and measuring D_3_-enrichment in urine creatinine within 48 to 96 h, one can determine the oral label’s dilution in the whole-body creatine pool. Since the creatine pool is strongly associated with skeletal muscle function, and not related to the muscle’s non-contractile components [48], this method can provide an accurate indicator of functional muscle mass. Moreover, D_3_-Cr can be used in large studies with minimal burden. 

### 4.2. Strengths and Limitations

To our knowledge, this is the first systematic review examining the effect of exercise specifically on sarcopenia among cancer survivors, and sarcopenia is highly relevant to cancer outcomes. In addition to published studies, our work was strengthened by searching ongoing trials to ensure we included relevant studies. One limitation is the small number of high-quality clinical trials in this area (seven total, only four of which were randomized, and only one was the primary outcome of the RCT). Our inclusion criteria focused specifically on sarcopenia, so trials with muscle mass and/or muscle function endpoints without sarcopenia diagnosis were not included [49,50,51,52]. However, previous systematic reviews and meta-analyses have supported the protective role of exercise on muscle mass in cancer patients [16,17], which is compatible with our findings. Another limitation was the relatively short study durations (most were 16 days to 17 weeks long, only one was 6 months long), such that long-term effects of exercise could not be evaluated. Therefore, more high-quality, long-term, large randomized controlled trials assessing muscle quantity and function are necessary to further examine the effect of exercise on sarcopenia among cancer survivors. In addition, future systematic reviews on this area of research could include other databases, such as Embase and Scopus, to ensure comprehensive inclusion of relevant studies. We were not able to conduct quantitative data synthesis due to heterogeneity of the interventions, comparators and outcomes of included studies. As the literature in this area grows, future systematic reviews should also consider conducting meta-analysis when possible. 

## 5. Conclusions

In summary, a relatively small but significant protective effect of exercise for sarcopenia was seen in most of the studies, despite the short intervention periods. The exercise trials included patients with breast, gastric, prostate, liver or rectal cancer, all of whom received different treatments based on their diagnosis. If this beneficial effect is supported in larger trials, we could potentially identify cancer survivors at higher risk of adverse health outcomes by screening for sarcopenia and improve their prognosis through exercise interventions. Several ongoing randomized controlled trials [53,54,55] could provide additional valuable information. Studies are also needed to further test the interaction between aging and cancer treatment and explore the optimal structure and exercise dose that is most effective for reversing and/or preventing sarcopenia in patients with different types of cancer. 

## Figures and Tables

**Table 1:** Diagnostic criteria of sarcopenia ^a^.

Reference	Cut-Off Points	Measurements
Adams et al. [23]	SMI > 1 SD (Class I sarcopenia) or > 2 SD (Class II sarcopenia) below age- and sex-specific normative values	DEXA
Yamamoto et al. [24]	4 m GS < 0.8 m/s; Handgrip strength < 30 kg in male/< 20 kg in female; SMI <8.87 kg/m^2^ in male/< 6.42 kg/m^2^ in female	BIA, handgrip strength and GS
Dawson et al. [25]	ASMI < 7.26 kg/m^2^ (male only)	DEXA
Dieli-Conwright et al. [26]	ASMI < 5.45 kg/m^2^ (female only) BMI ≥ 30.0 kg/m^2^ (for sarcopenic obesity)	DEXA
Koya et al. [27]	SMI < 42 cm^2^/m^2^ in male/< 38 cm^2^/m^2^ in female	CT
Moug et al. [28]	TPI < 524 mm^2^/m^2^ in male/< 385 mm^2^/m^2^ in female	CT
Delrieu et al. [29]	SMI < 40 cm^2^/m^2^	CT

^a^ ASMI = appendicular skeletal muscle index; BIA = bioimpedance analysis; BMI = body mass index; CT = computer tomography; DEXA = dual-energy X-ray absorptiometry; GS = gait speed; SD = standard deviation; SMI = skeletal muscle index; TPI = total psoas index.

**Table 2:** Baseline characteristics of participants ^a^.

Reference	Study Type	Sample Size	Age (Year) ^b^	Female (%)	Cancer Type	Treatment	Sarcopenia (%)
Adams et al. [23]	RCT	200	48.8 (25.0–78.0)	100	Breast cancer (Early stages: I to IIIa)	Receiving adjuvant chemotherapy	25.5 (25.0% class I; 0.5% class II)
Yamamoto et al. [24]	Quasi-experimental	22	75.0 ± 5.0	54.5	Gastric cancer (All stages)	Scheduled gastrectomy (pre-operative)	100
Dawson et al. [25]	Pilot RCT	37	63.7 ± 8.3	0	Prostate cancer (All stages)	Receiving ADT	43.8
Dieli-Conwright et al. [26]	RCT	100	53.5 ± 10.4	100	Breast cancer (Early stages: I to III)	Completed treatment < 6 months (chemotherapy and/or radiotherapy)	Not reported
Koya et al. [27]	Quasi-experimental	209	74.7 (69.0–79.6) ^c^	35.4	HCC (All stages)	Treated with TACE	Not reported
Moug et al. [28]	RCT	44	66.8 ± 9.6	36	Rectal cancer (Stage not reported)	Receiving neoadjuvant chemoradiotherapy	14
Delrieu et al. [29]	Quasi-experimental	47	55 ± 10.4	100	Breast cancer (Metastatic)	Receiving any combination of chemotherapy, radiotherapy, hormonal therapy and targeted therapy	53.2

^a^ ADT = androgen deprivation therapy; HCC = hepatocellular carcinoma; NA = not applicable; RCT = randomized clinical trial; TACE = transcatheter arterial chemoembolization. ^b^ Unless otherwise stated, ages are presented as mean ± standard deviation or mean (range). ^c^ Median (IQR).

**Table 3:** Study design and results ^a.^

Reference	Intervention	Duration	Adherence	Results	△Muscle Index ^d^		Sarcopenia Reversed ^d^
					Vs. BL	Vs. Control	
Adams et al. [23]	Three times/week; Supervised RET or AET	17 weeks ^b^	RET: 68.2% AET: 72.0%	(1) Increased SMI: △SMI between PI and BL in AET = 0.21 (0.01–0.05) kg/m2; △SMI between PI and BL in RET = 0.36 (0.17–0.55) kg/m2; Difference in △SMI between RET and UC = 0.32 (0.04–0.60) kg/m2, *p* = 0.017; Difference in △SMI between AET and UC = 0.18 (−0.10–0.46) kg/m2, *p* = 0.35 (2) Reversed sarcopenia: RET vs. UC/AET: 42.9% vs. 16.7%, *p* = 0.039	2.4% ^e^	2.1% ^e^	26.2%
Yamamoto et al. [24]	Daily; Unsupervised Combined RET and AET and nutritional support	16 days ^b^	50%	(1) Handgrip strength increased: PI vs. BL: 21.2 ± 5.2 kg vs. 20.0 ± 5.3 kg, *p* = 0.022 (2) Gait speed increased (not significant): PI vs. BL: 0.85 ± 0.22 m/s vs. 0.80 ± 0.21 m/s, *p* = 0.06 (3) SMI increased (not significant): PI vs. BL: 6.22 ± 0.70 kg/m^2^ vs. 6.12 ± 0.69 kg/m^2^, *p* = 0.06 (4) Sarcopenia reversed: 4 patients (18.2%) became nonsarcopenic after the program	1.6%	NA	18.2%
Dawson et al. [25]	Three times/week; Supervised RET; With or without protein supplementation	12 weeks	>85%	(1) Attenuated sarcopenia prevalence: EXE vs. NoEXE ^c^:—23.1% vs. + 5.2%, *p* = 0.04 (2) Increased ASMI in EXEc group: △ASMI between PI and BL = 0.3 kg/m^2^, 95% CI: 0.2–0.4 (3) Better ASMI change in the exercise groups: EXE vs. NoEXE ^c^: 3.6% vs. 0.1%, *p* = 0.02 Difference of △ASMI between EXE and NoEXE = 0.3 kg/m^2^, *p* = 0.02	3.6%	3.7%	28.3%
Dieli- Conwright et al. [26]	Three times/week; Supervised Combined RET and AET	16 weeks	95%	(1) Increased LBM in the intervention group: BL vs. PI LBM: 53.8 ± 7.9 vs. 56.7 ± 8.0 (kg), *p* = 0.001 (2) PI LBM higher in the intervention group: Exercise vs. UC: 56.7 ± 8.0 vs. 49.0± 7.9 (kg), Difference in PI LBM = 7.7 kg, 95% CI: 5.5–10.3 kg, *p* = 0.001 (3) Increased ASMI in the intervention group: BL vs. PI SMI: 5.0 ± 0.4 vs. 6.6 ± 0.6 (kg/m^2^), *p* = 0.001 (4) PI ASMI higher in the intervention group: Exercise vs. UC: 6.6 ± 0.6 vs. 4.2 ± 0.4 (kg/m^2^), Difference in PI ASMI = 2.4 kg/m^2^, 95% CI: 1.3–4.1 kg/m^2^, *p* = 0.001	5.4 % (LBM); 32% (ASMI)	12.8 % (LBM); 50% (ASMI)	Not reported
Koya et al. [27]	Daily; In-hospital Combined RET and AET	52 days ^b^	Not reported	(1) Higher △SMI change in the exercise group: *p* < 0.001 (2) Exercise is an independent factor for an increase in SMI: HR = 2.13 (1.215 ~ 3.846), *p* = 0.009	Not reported	Not reported	Not reported
Moug et al. [28]	Daily; Unsupervised AET	14 weeks	Not reported	(1) Increased median TPI in the intervention group: Crude: intervention vs. control: +16.0 vs. −8.4 mm/m^2^, *p* = 0.07 Adjusted: mean difference = 40.2 mm^2^/m^2^, 95% CI: −3.4 to 83.7 mm^2^/m^2^, *p* = 0.07	2.7%	6.9% ^f^	Not reported
Delrieu et al. [29]	Daily; Unsupervised AET	6 months	Not reported	(1) No significant change in cross sectional muscle area, SMD, LBM and SMG (*p* = 0.75, 0.07, 0.75 and 0.06) (2) Improvement in 6 min walking distance: +7%, *p* < 0.001 (3) Improvement in isometric quadriceps strength: +22%, *p* < 0.001	Not reported	Not reported	Not reported

^a^ AET = aerobic exercise training; ASMI = appendicular skeletal muscle index; BL = baseline; BMI = body mass index; GS = gait speed; HR = hazard ratio; LBM = lean body mass; NA = not applicable; PI = post-intervention; RET = resistance exercise training; SMD = skeletal muscle radiodensity; SMI = skeletal muscle index; SMG = skeletal muscle gauge; TPI = total psoas index; UC = usual care group; △ = change between baseline and post-intervention. ^b^ Median intervention time. ^c^ Groups with an exercise segment compared with groups without exercise segment. ^d^ Calculated using data reported by publications. Muscle mass index including SMI, ASMI and TPI. △Muscle mass index vs. BL = difference between post- and pre-intervention muscle mass index mean in intervention group/pre-intervention muscle mass index mean in the intervention group; △Muscle mass index vs. control = difference between the change in muscle mass index mean in intervention and control groups at the end of the study/pre-intervention muscle mass index mean in the intervention group; Sarcopenia reversed = difference of proportion of sarcopenia being reversed after intervention in intervention and control groups (for Adams et al.: RET vs. AET/UC), or (for Yamamoto et al.) proportion of sarcopenia being reversed after intervention in the intervention group. ^e^ Data in RET group was used. ^f^ Adjusted group difference was used.
